# Supplementary material for: Automatic Differentiation is no Panacea for Phylogenetic Gradient Computation
Source: Genome Biol Evol. 2023 Jun 2;15(6):evad099. doi: 10.1093/gbe/evad099 (PMC10282121; doi:10.1093/gbe/evad099)
Supplement: evad099_Supplementary_Data [file evad099_supplementary_data.pdf]

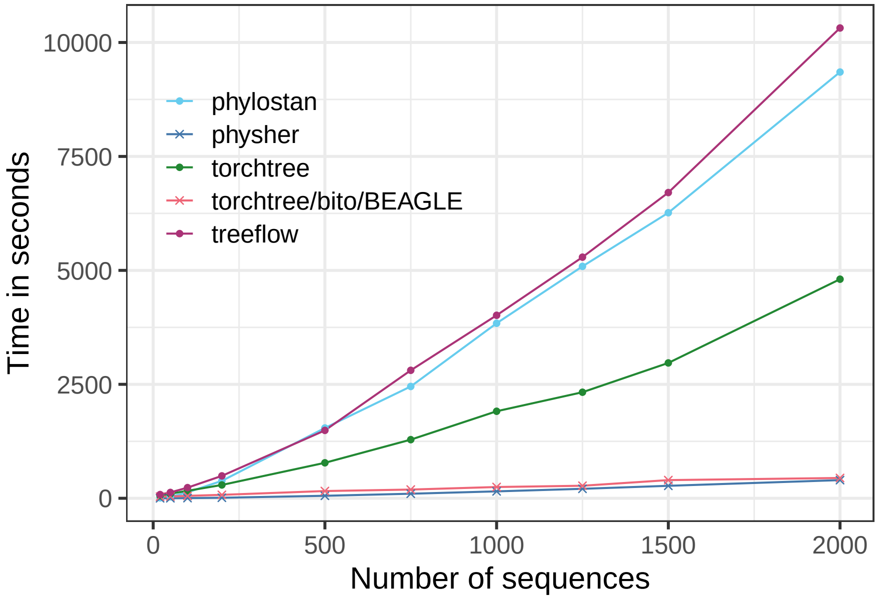

Figure S1: Speed of implementations for 5000 iterations of variational time-tree inference with a strict clock.

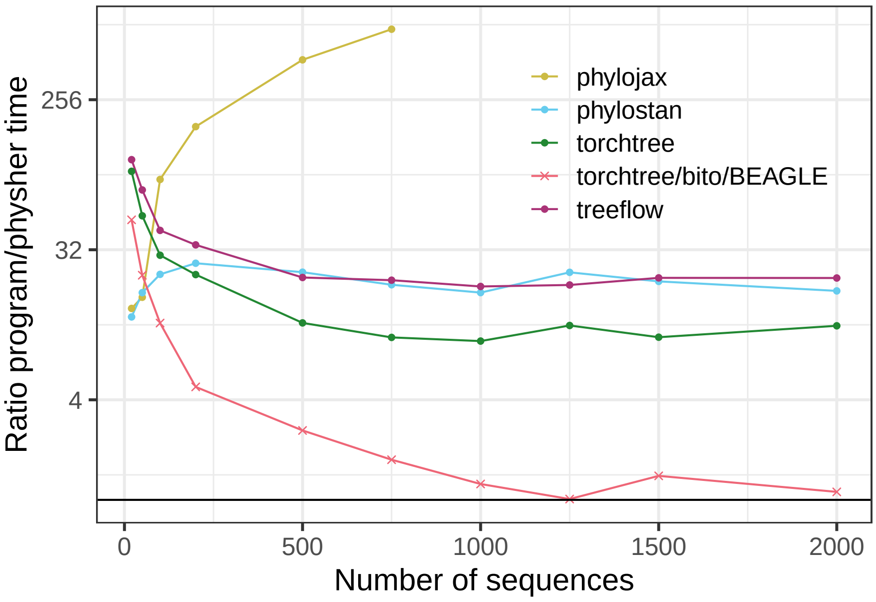

Figure S2: Relative performance of each implementation against `physher`. The black horizontal line intersects the y-axis at 1.

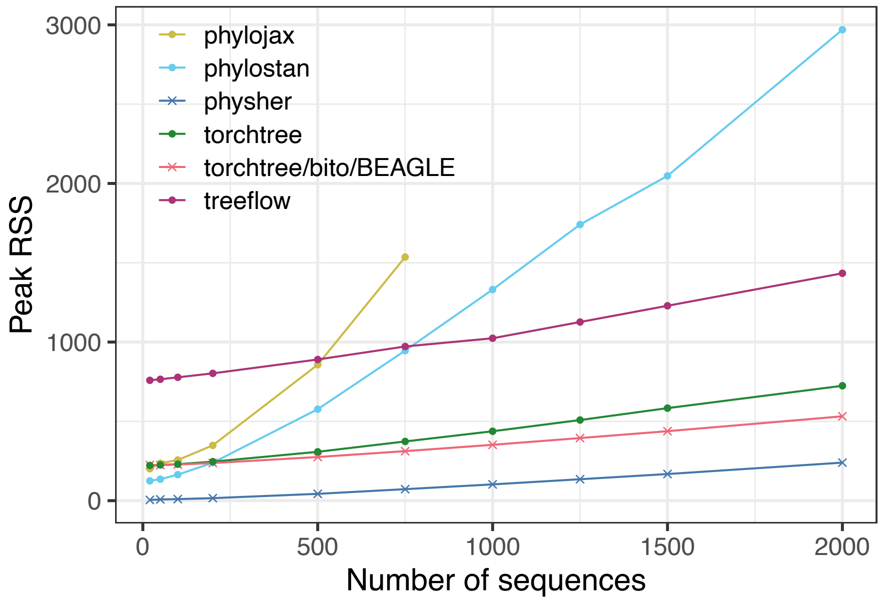

Figure S3: Peak RSS (resident set size) memory usage of implementations for 5000 iterations of variational time-tree inference with a strict clock.

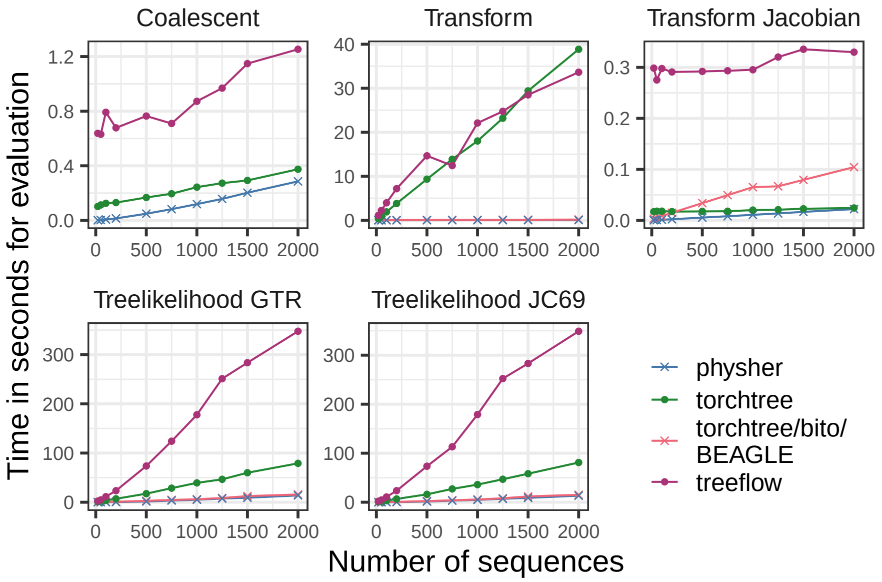

Figure S4: Speed of implementations for the evaluation component for various inferential tasks. See text for description of the tasks. `phylojax` results are excluded from this plot; see Figure S6 for `phylojax`. See Figure S6 for function evaluations.

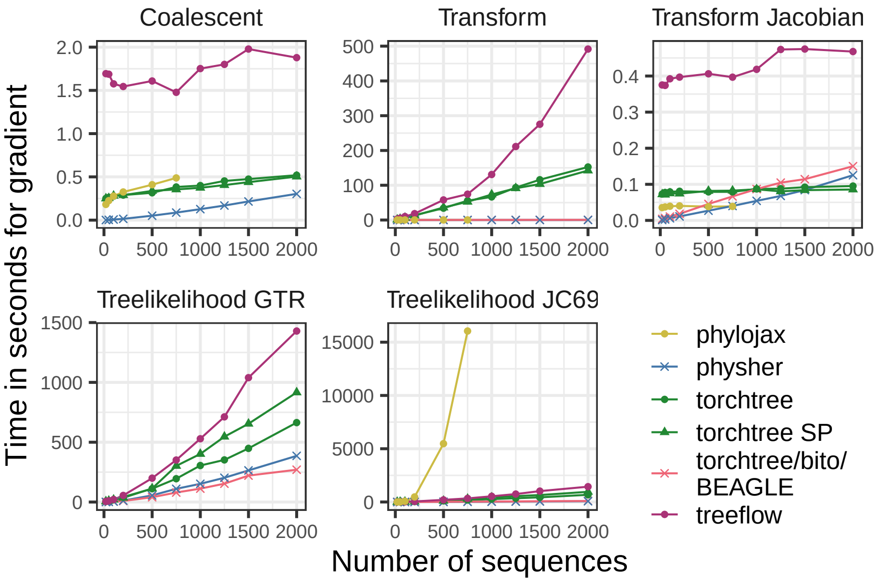

Figure S5: Speed of implementations for the gradient component for various inferential tasks. The `torchtree SP` label denotes `torchtree` running with single precision. Just-in-time compilation is enabled for `phylojax`.

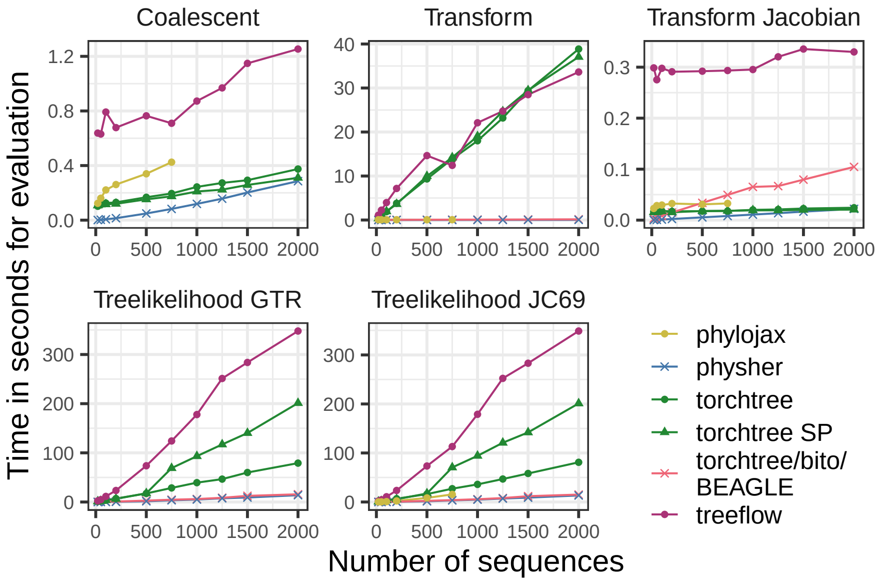

Figure S6: Speed of implementations for the evaluation component for various inferential tasks. The torchtree SP label denotes `torchtree` running with single precision. Just-in-time compilation is enabled for `phylojax`.

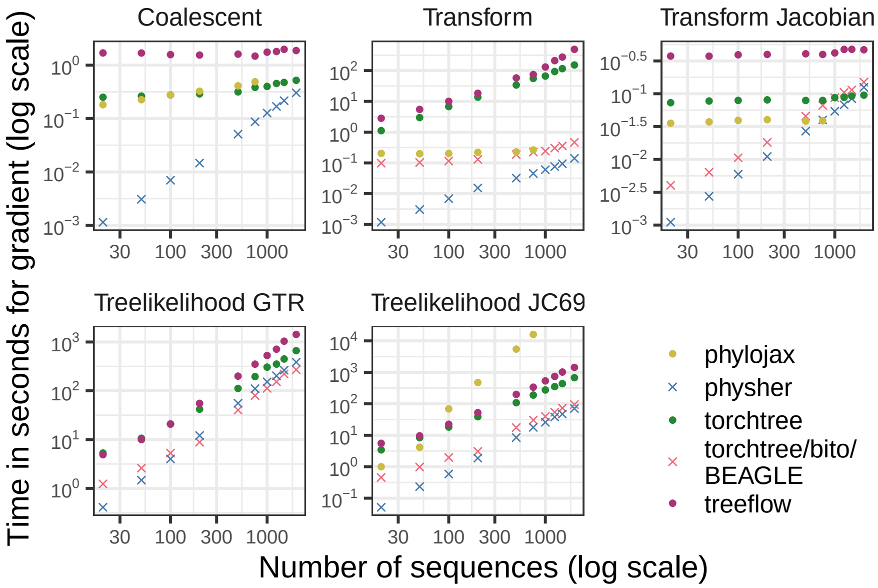

Figure S7: Log-log plot of gradient calculation time against dataset size for various inferential tasks. Just-in-time compilation is enabled for `phylojax`.

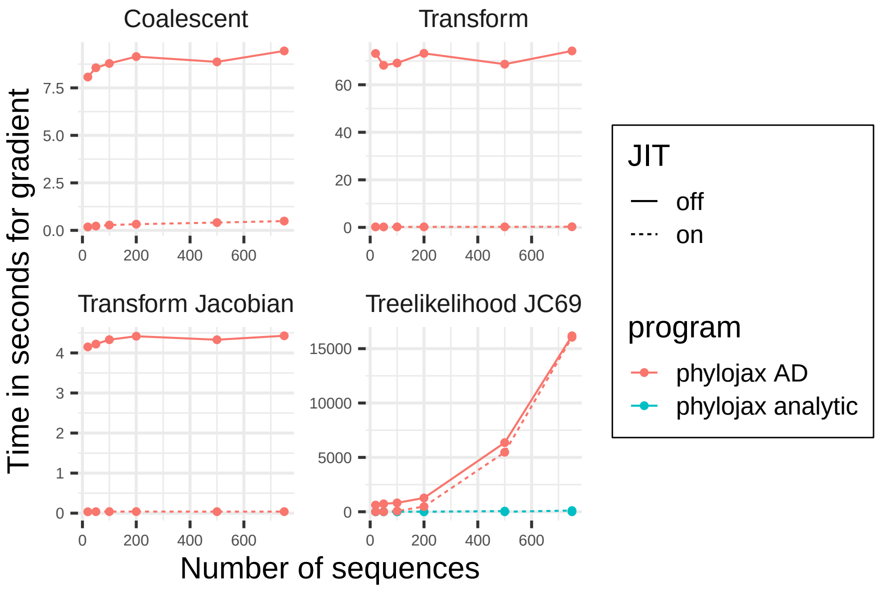

Figure S8: Gradient calculation time against dataset size for various inferential tasks with `phylojax`. Gradient are calculated using automatic differentiation (AD) or finite differences. Just-in-time (JIT) compilation is either turned on or off.
